# Lower Lip Suspension With Gore-Tex Suture: Technique and Literature Review

**Published:** 2014-10-08

**Authors:** John Paul Tutela, Jared Davis, Matthew Zeiderman, Sharooz Sean Kelishadi, Bradon Wilhelmi

**Affiliations:** Division of Plastic and Reconstructive Surgery, University of Louisville School of Medicine, Louisville, Ky

**Keywords:** oral incompetence, lip suspension, free-flap reconstruction, Gore-Tex suture, oral carcinoma

## Abstract

**Objective:** Oral incompetence is a problem frequently experienced after free-flap reconstruction of the oral cavity for patients with oral carcinoma. We describe an interesting treatment modality to deal with this pathology. **Methods:** A 60-year-old woman diagnosed with squamous cell carcinoma of her oral floor was treated with chemoradiation with complete response, and after suffering recurrence underwent composite mandibulectomy via visor flap and immediate fibular free flap reconstruction. Postoperatively, she was treated with adjuvant chemoradiation and developed oral incompetence months afterward. We performed lower lip suspension with Gore-Tex (Gore – Flagstaff, Arizona) suture with good functional and aesthetic outcome. **Results:** As of 9 months postoperatively, oral competence was achieved with good functional and aesthetic outcome. **Conclusions:** This approach is a viable, simple means of restoring oral competence secondary to loss of static control of the lower lip.

Surgery is the prevailing mode of treatment for squamous cell carcinoma of the oral cavity. It has the best prognosis and rate of local control for oral floor cancer.^1^ These tumors often invade surrounding structures including the base of the tongue and the mandible, and these patients often require composite resection including partial glossectomy and partial mandibulectomy. Taylor et al first described the free tissue transfer of vascularized fibula in 1975, and it was later reported as an alternative for mandibular reconstruction following oral tumor resection by Hidalgo.^2^^,^^3^ Since then, it has become a popular method of mandibular reconstruction due to its bone length, ease of elevation on a supine patient undergoing oral surgery, the vascular nature of its periosteum, the reliability of the septocutaneous perforators, and its suitability for repairing composite defects. However, like any other intervention, it has potential complications. Hidalgo reported a 10-year follow-up of his patient cohort, and while donor site morbidity was minimal, the 75% experienced oral incontinence. Of note, 10% of this group received radiation preoperatively, 60% postoperatively, and oral incontinence was postulated to be secondary to altered mental nerve sensation. However, those who received radiation were also found to have higher rates of asymmetry and functional deficit.^4^

## METHODS

Our patient was a 60-year-old Caucasian woman who sought medical attention in March 2009 for evaluation of a lesion of her oral floor. It had been present for several weeks and temporally associated with intolerance for solids and weight loss. She was diagnosed with squamous cell carcinoma, declined operative treatment, and subsequently underwent Cisplatin chemotherapy and 7200 centigray of external beam radiation. Although thought to have a complete response, her disease recurred by January 2010 and she was referred for surgical evaluation. She subsequently underwent composite mandibulectomy from angle to angle and near-total glossectomy. The right marginal mandibular nerve was sacrificed because of tumor involvement. Immediate reconstruction was performed with fibular free-flap with plate fixation to each ipsilateral ramus and in the midline. Adjuvant therapy was with carboplatin, paclitaxol, and 5700 centigray external beam radiation. Although the patient had good salivary continence in the immediate postoperative period, 25 months following adjuvant therapy, she developed a fibrotic response and loss of static control of her lower lip, oral incompetence, impaired facial expression, and dysarthria. This posed a major functional and social impediment, and she sought reconstruction.

**Figure 1:**
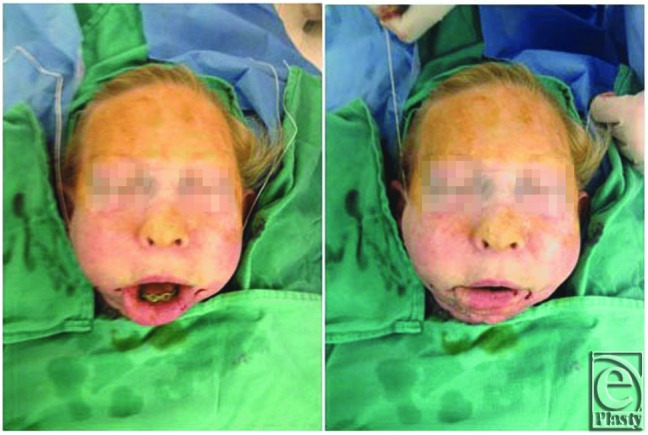
Correction of oral incompetence demonstrated by tightening of the Gore-Tex suture sling.

We provided static sling suspension of the lower lip with 2 Gore-Tex sutures. A suture passer was used to pass 2 Gore-Tex sutures from the right temporal fossa, through the preauricular region, to the right oral commissure, and through the white roll of the lip. The sutures were then tunneled up the left side along the same course and secured to the left temporalis fascia. After placing appropriate tension, the sutures were secured to the right temporalis fascia. The lip was overcorrected to allow for tissue relaxation and prosthetic stretch without compromise of function (Fig 1).

## RESULTS

She had no immediate complications and static function of the lower lip had been restored with return of oral competence. As of 9 months postoperatively, the result was still durable, without infectious or inflammatory complications, and the patient reported further improvement in glutition, speech, and salivary continence.

## DISCUSSION AND LITERATURE REVIEW

An increasing experience with reconstruction after treatment of oral cavity malignancies has presented the additional challenge of how to deal with the resulting complications. While there is not much literature on revision of such operations specifically, much can be derived from historic classification of oral sphincteric dysfunction and methods of repair. The lips are the main aesthetic component of the lower face, and their function is vital to oral competence, swallowing, facial animation, and speech.^5^^-^7 Dysfunction and functional impairment result in significant social problems. Classically, deficits in oral sphincter function are considered static or dynamic in nature. Dynamic function is the ability to animate the mouth, lips, and cheeks. Static control refers to the ability to keep the mouth symmetrically closed. Historically, it is believed that static function of the upper lip in the presence of good lower lip control is sufficient for oral competence, and many approaches have been described to provide lower lip suspension and correct oral incompetence. These approaches can be divided into autologous versus prosthetic, and autologous methods can be further subdivided into microneurovascular grafts and local rearrangement. Restoration of dynamic function is more complex and often utilizes microneurovascular flaps or nerve grafting. Local muscle transfer using the temporalis or masseter are alternatives. Static function can be effectively restored with a fascial, tendinous, or synthetic sling and our discussion will focus on these methods.

Fascial and tendon sling grafts are one of the most well-described methods of static repair. Multiple variations of a forearm flap with palmaris longus tendon graft for static suspension of the lower lip have been discussed in the literature. Sakai and colleagues described a composite radial forearm flap with palmaris longus tendon graft to provide static suspension of the reconstructed lower lip in a patient with a total lower lip and jaw defect in 1989. This procedure utilized the forearm donor skin as an external skin and mucosal lining for the defect. The palmaris longus tendon was anchored to the modiolus bilaterally and served as a static sling.^8^ Jeng et al^9^ modified this technique with good result for patients requiring resection of various malignancies of the lip and cheek. This alteration sought to provide more dynamic support and better oral competence by folding the tissue flap over the palmaris longus tendon, bilaterally passing the tendon intramuscularly through modiolus at the angles of the mouth and anchoring the graft to the orbicularis oris near the philtrum.^9^ This successfully achieved oral competence at rest and during speech and mastication in 2 additional studies.^10^^,^^11^ Carroll et al also demonstrated good results in their series of 10 patients with total lip and chin defects, all of whom underwent radial forearm and palmaris longus tendon free flap.^10^ Fernandes and Clemow^11^ reported good results for 13 patients with full-thickness lip defects. Their cohort included posttraumatic defects and patients who underwent surgical treatment of cutaneous lip malignancy; 10 patients who had resection of cutaneous malignancies of the lip and 8 whom received adjuvant or neoadjuvant radiation. With 1 exception, an anterolateral thigh flap, all were reconstructed with radial forearm flap using palmaris tendon. Satisfaction was graded by the patients, and all were reported to tolerate oral diet, and have oral competence, although the length of follow-up was not reported, and it is difficult to determine the durability of this approach. Solitary palmaris longus tendon, without the use of vascularized free tissue transfer, has also been described to provide static lower lip suspension in the setting of facial palsy without tissue deficit.^12^

Use of plantaris tendon has also been described. Yoleri et al described splicing the tendon to create a trivectorial suspension anchored at the modiolus and attached to preauricular and temporal fascia. This patient cohort included individuals with facial paralysis and resultant asymmetry and oral sphincter dysfunction. The tendon did not develop laxity or allow nasolabial droop at average follow-up of 17 months, thus demonstrating durability and viability for static suspension.^13^ While significant stretch did not occur, loosening of the tendon at its fixation sites to the temporal fascia was reported.

Both plantaris and palmaris are of appropriate length, width, and tensile strength for static or dynamic suspension procedures. Both had low donor site morbidity; however, a significant shortcoming is that each is absent bilaterally in 5% to 15% of people.^14^^,^^15^ While absence of palmaris is detectable on physical examination, the same is not true for plantaris. Furthermore, the tendons are not always suitable for grafting due to insufficient length suitability for grafting.^16^

Fascial sling use has also been reported. The muscle bow traction method utilizes a tensor fascia lata sling attached to the orbicularis oris and bowed through the masseter muscle to provide tension and movement to the lips and mouth to correct oral incompetence and facial palsy. With this method, Maegawa et al^17^ demonstrated the successful correction of salivary incontinence and enhanced oral commissure movement in a small number of patients who presented with facial palsy 1 to 3 years after underdoing unilateral parotidectomy or resection of a cerebellopontine angle schwannoma.

In addition to fascial and tendon sling methods of repair, local muscle flaps are also well described. Chan et al^18^ described suspension of the lower lip using bilateral temporalis muscle flaps in combination with a fascia lata sling to successfully correct oral incompetence. Other groups have also achieved satisfactory outcomes with a similar procedure utilizing temporalis flaps, both with and without a fascia lata extension to the orbicularis oris.^19^^,^^20^ One series described good results using temporalis tendon transfer re-animation in patients with long-standing facial paralysis.^19^ As opposed to using the temporalis muscle flap, which often leaves bulk over the zygomatic arch, masseter muscle flaps have been tethered to fascia lata slings, which utilize the zygomatic arch as a trochlea to restore oral competence and facial animation to the lips and mouth.^21^

While all of the aforementioned methods use autologous tissue, the use of synthetic materials has also shown promise. Gore-Tex strips provided static suspension in a series of 17 patients who lacked suitable autologous graft immediately following total parotidectomy and facial nerve resection.^22^ This approach sutured the bifurcated inferior end of a 15-mm strip to both the upper and lower lip near the modiolus, and the supportive superior ends of the strip to the zygomatic arch. This retrospective cohort study reported good short-term outcomes. Long-term complications include infection, sling extrusion, and chronic inflammation. All patients in the study underwent neoadjuvant or adjuvant radiation therapy, which may have contributed to long-term complications.^22^ In a different study, long-term efficacy was achieved in 31 of 32 patients using Gore-Tex strips to provide static suspension of the lip in patients with facial nerve paralysis shortly after extirpative skull base surgery.^23^ All patients in this study achieved oral competence as well as satisfactory aesthetic outcomes and only 2 required revisions. On the other hand, a Gore-Tex single strip anchored to the malar eminence and bifurcated to attach to the orbicularis at the nasolabial and melolabial folds yielded less desirable outcomes in multiple patients with facial paralysis. Complications included sling failure in 1 patient and sling laxity in the remaining patients between 3 to 9 months postoperatively.^24^ Moreover, simple Gore-Tex sutures can be used for static facial suspension as well. Gore-Tex sutures were used successfully without complications for bilateral static suspension of the anterior mid-face and accompanying cosmetic procedures in 187 of 197 cases, and only 1 case required suture removal secondary to infection 3 months postoperatively.^25^

Thus, while the long-term efficacy of Gore-Tex strip static sling suspension remains to be determined and variability in procedure methods and patient populations somewhat complicate comparisons, both Gore-Tex strips and cable sutures appear to demonstrate satisfactory outcomes and durability for static suspension of facial musculature and restoration of oral competence when utilized under proper circumstances.^23^^-^26 Furthermore, given the demonstrated strength and nonimmunogenic nature of Gore-Tex, it may ultimately prove an acceptable substitute for patients who require static suspension but are unable to undergo autologous reconstruction.^27^^-^29

We initially sought to perform plantaris sling suspension; however, our patient was missing plantaris muscle and tendon bilaterally. Palmaris tendon length was inadequate for bilateral repair. A remaining alternative was use of an extensor digitorum longus tendon. However, this procedure had not been discussed with the patient in advance and would have exposed her to more risk than was originally agreed upon with the patient. Furthermore, a sufficiently long extensor tendon cannot be harvested with a tendon stripper due to the extensor retinacula. Age-related cartilaginous fusion of the extensor tendons poses another likely obstacle. In addition, our patient had preexisting gait disturbances, and this operation may have been more morbid in this setting. The use of a temporalis transfer as previously described was also an undesirable alternative given our patient's surgical history. Furthermore, the temporalis transfer likely would have left a bulge over the lateral orbital margin as well, creating another facial deformity. Masseter transfer was ruled out as an option due to further donor-site morbidity. Therefore, Gore-Tex sutures were chosen intraoperatively as an alternative solution.

While other authors have reported the use of Gore-Tex sutures for immediate static suspension in patients with facial paralysis, this is the first description of delayed static lower lip suspension with Gore-Tex suture following composite mandibulectomy in a patient who developed oral incompetence after adjuvant external beam radiotherapy. Moreover, our method utilizes a continuous sling as opposed to multiple strips or sutures. The temporalis fascia functions as a suitable anchor in a patient such as ours who lacks the natural muscular support around the zygomatic arch as a consequence of mandibulectomy. Lacking this natural support, we felt a continuous sling anchored bilaterally to the temporal fascia was appropriate, and less invasive than free tissue transfer or local rearrangement.

Our patient had many key differences when compared with the other clinical scenarios discussed that rendered our technique appealing and successful. Having undergone free flap reconstruction of the lip and mandible, she lacked significantly more soft and bony tissue than a patient who has undergone parotidectomy, resection of cutaneous malignancy, or isolated facial paralysis. Although our patient had subtotal glossectomy, bilateral mental nerve, and unilateral marginal mandibular nerve sacrifice at the time of tumor extirpation, she initially did have salivary continence. This changed 25 months after completion of adjuvant radiation with a loss of salivary continence. Consequently, we believe the radiation contributed by induction of fibrosis and inflammation, and neuritis ultimately set in.

The risk of suture breakage or disconnection from the temporal fascia is sufficiently low in a patient such as ours: an individual with altered facial muscle structure is unlikely to expose the sutures to the same physiological stress as someone with an intact mandible and facial musculature. Furthermore, our patient retained dynamic control of her upper lip.

## CONCLUSIONS

Many procedures for the correction of salivary incontinence and facial nerve paralysis have been well documented; however, success of these procedures is variable and long-term efficacy has not been published for most. Comparison of techniques is complicated by variability in patient population. The long-term efficacy of our approach relative to other solutions for correcting oral incompetence and providing static lower lip-suspension remains to be determined. Future outcome reports using this method would help determine its longevity.
